# 2-[(2-Chloro­phen­yl)(hy­droxy)meth­yl]phenol

**DOI:** 10.1107/S1600536813018667

**Published:** 2013-07-13

**Authors:** G. Prakasha, Jerry P. Jasinski, Jared S. Brown, H. S. Yathirajan, D. K. Ravishankara

**Affiliations:** a Department of Studies in Chemistry, University of Mysore, Manasagangotri, Mysore 570 006, India; b Department of Chemistry, Keene State College, 229 Main Street, Keene, NH 03435-2001, USA; c Sri Mahadeshwara Government First Grade College, (Affiliated to University of Mysore), Kollegal 571 440, India

## Abstract

In the title compound, C_13_H_11_ClO_2_, the dihedral angle between the mean planes of the 2-chloro­phenyl and phenol rings is 87.4 (9)°. The methyl hy­droxy group lies nearly perpendicular to the plane of its attached benzene ring [O—C—C—C torsion angle = 84.3 (3)°]. The two hy­droxy groups lie on the same side of the mol­ecule and are in a slightly twisted gauche conformation [O—C—C—O torsion angle = 77.1 (8)°] to each other. In the crystal, O—H⋯O hydrogen bonds between nearby methyl­hydroxy groups form dimers in alternating pairs aligned diagonally along the *b* axis. A view along the *c* axis reveals a hexa­meric aggregate mediated by a ring of six O—H⋯O hydrogen bonds generating an *R*_6_^6^(12) motif loop.

## Related literature

For general background to the use of benzhydrols, see: Ohkuma *et al.* (2000 ▶). For the use of the title compound in the perfume and pharmaceutical industries, see: Meguro *et al.* (1985 ▶). For related di­phenyl­methanol structures, see: Betz *et al.* (2011 ▶); Ferguson *et al.* (1995 ▶); Siddaraju *et al.* (2010 ▶).
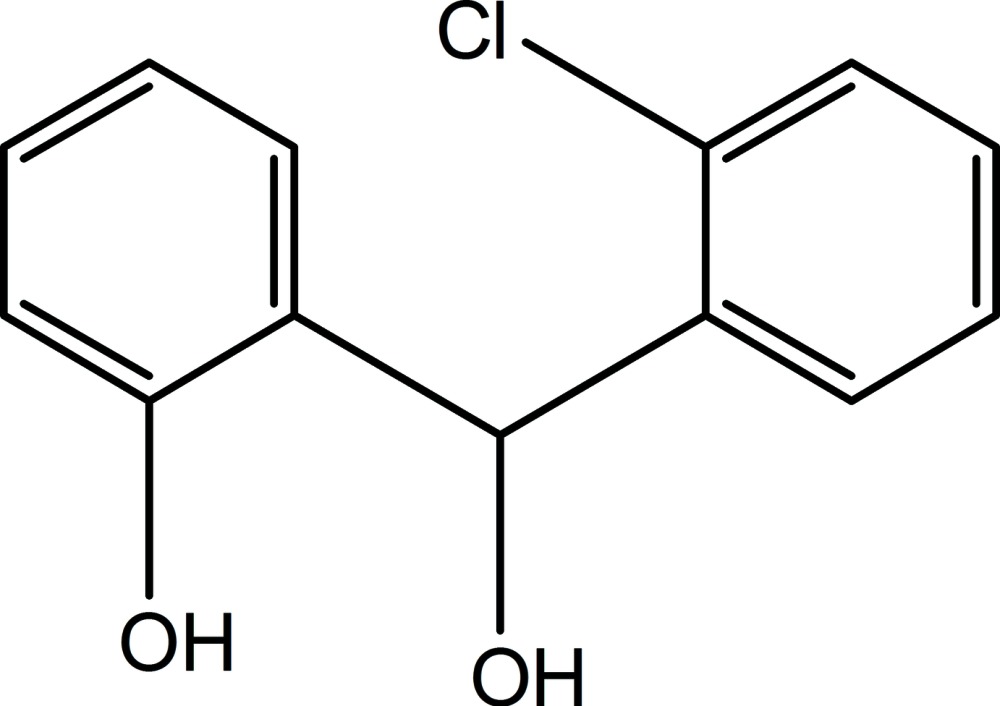


## Experimental

### 

#### Crystal data

C_13_H_11_ClO_2_
*M* *_r_* = 234.67
Trigonal, 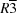

*a* = 23.4627 (8) Å
*c* = 11.3722 (4) Å
*V* = 5421.6 (4) Å^3^
*Z* = 18
Cu *K*α radiation
μ = 2.66 mm^−1^
*T* = 173 K
0.46 × 0.38 × 0.24 mm

#### Data collection

Agilent Xcalibur (Eos, Gemini) diffractometer
Absorption correction: multi-scan (*CrysAlis PRO* and *CrysAlis RED*; Agilent, 2012 ▶) *T*_min_ = 0.517, *T*_max_ = 1.000
11532 measured reflections
2364 independent reflections
2055 reflections with *I* > 2σ(*I*)
*R*_int_ = 0.047

#### Refinement

*R*[*F* ^2^ > 2σ(*F* ^2^)] = 0.065
*wR*(*F* ^2^) = 0.213
*S* = 1.07
2364 reflections
151 parameters
H atoms treated by a mixture of independent and constrained refinement
Δρ_max_ = 0.40 e Å^−3^
Δρ_min_ = −0.62 e Å^−3^

### 

Data collection: *CrysAlis PRO* (Agilent, 2012 ▶); cell refinement: *CrysAlis PRO*; data reduction: *CrysAlis RED* (Agilent, 2012 ▶); program(s) used to solve structure: *SUPERFLIP* (Palatinus & Chapuis, 2007 ▶); program(s) used to refine structure: *SHELXL2012* (Sheldrick, 2008 ▶); molecular graphics: *OLEX2* (Dolomanov *et al.*, 2009 ▶) and *Mercury* (Macrae *et al.*, 2006 ▶); software used to prepare material for publication: *OLEX2*.

## Supplementary Material

Crystal structure: contains datablock(s) global, I. DOI: 10.1107/S1600536813018667/sj5343sup1.cif

Structure factors: contains datablock(s) I. DOI: 10.1107/S1600536813018667/sj5343Isup2.hkl

Supplementary material file. DOI: 10.1107/S1600536813018667/sj5343Isup3.cml

Additional supplementary materials:  crystallographic information; 3D view; checkCIF report

## Figures and Tables

**Table 1:** Hydrogen-bond geometry (Å, °)

*D*—H⋯*A*	*D*—H	H⋯*A*	*D*⋯*A*	*D*—H⋯*A*
O1—H1⋯O1^i^	0.84	1.84	2.656 (2)	163

Symmetry code: (i) 
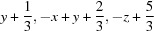
.

**Fig. 1.**
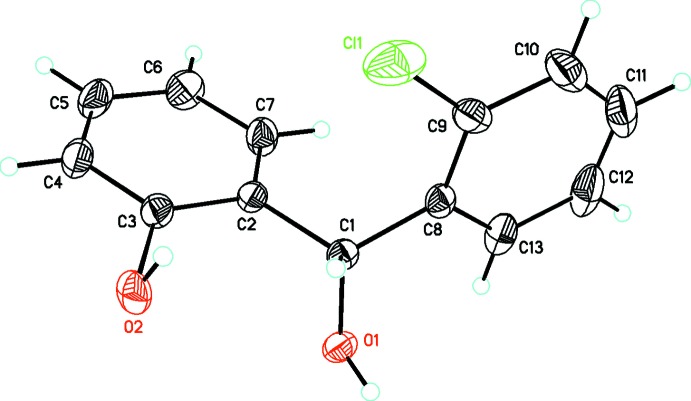
Molecular structure of the title compound showing the atom labeling scheme and 30% probability displacement ellipsoids.

**Fig. 2.**
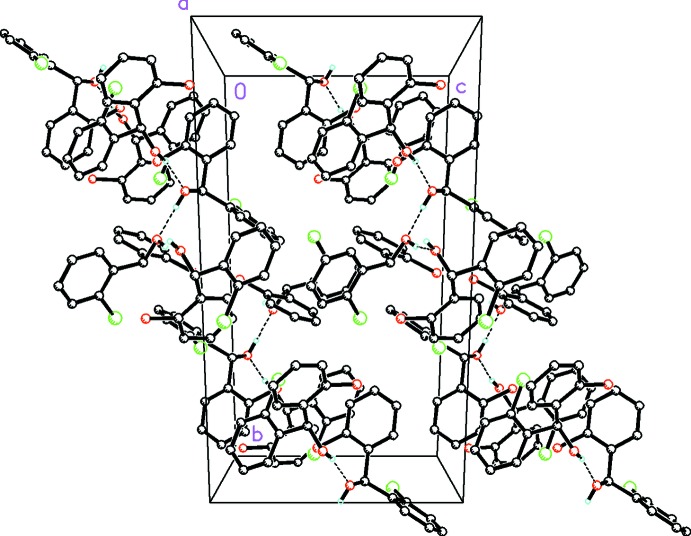
Packing diagram of the title compound viewed along the a axis. Dashed lines indicate O1—H1···O1 hydrogen bonds between nearby methylhydroxy groups which form dimers in alternating pairs algned diagonally along the b axis. H atoms not involved in hydrogen bonding have been deleted for clarity.

**Fig. 3.**
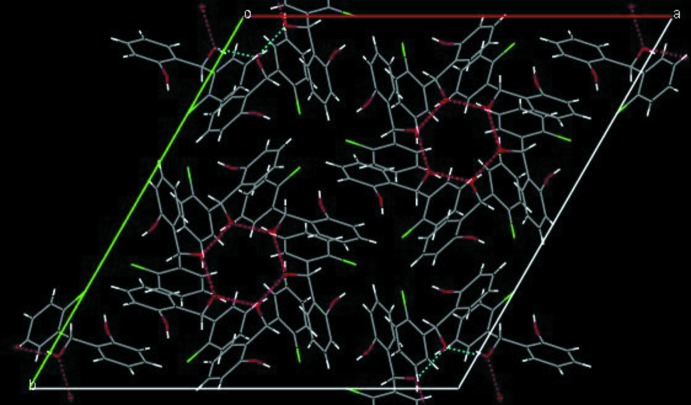
Packing of the molecules along c axis displaying a hexameric aggregate mediated by a ring of six O—H···O hydrogen bonds generating an R66(12) motif loop.

## supplementary crystallographic information

### Comment

Optically active diarylmethanols are crucial structural elements in many physiologically or/and biologically active molecules such as in the antihistamines (R)-orphenadrine and (S)-neobenodine. Benzhydrols are widely used as intermediates for the synthesis of pharmaceuticals (Ohkuma *et al.*, 2000), including drugs such as diphenhydramine, orphenadrine, diphenidol and phenyltoloxamine. The crystal structures and hydrogen bonding in some diphenylmethanols have been reported (Ferguson *et al.*, 1995). The title compound, 2-[(2-chlorophenyl) hydroxymethyl]phenol, (I), is a derivative of diphenylmethanol and it is used in the perfume and pharmaceutical industries (Meguro *et al.*, 1985). Recently, the crystal structure of (2-methylphenyl) (phenyl)methanol (Siddaraju *et al.*, 2010) and 2-(2-benzylphenyl) propan-2-ol (Betz *et al.*, 2011) were reported. In view of the importance of diarylmethanols, this paper reports the crystal structure of the title compound, C_13_H_11_O_2_Cl, (I).

In (I), the dihedral angle between the mean planes of the 2-chlorophenyl and phenol rings is 87.4 (9)° (Fig. 1). The methyl hydroxy group lies nearly perpendicular to the plane of its attached benzene ring [O1/C1/C2/C3 torsion angle = 84.3 (3)°]. The two hydroxy groups lie on the same side of the molecule and are in a slightly twisted gauche conformation [O1/C1/C3/O2 angle = 77.1 (8)°] to each other. In the crystal O—H···O hydrogen bonds between nearby methylhydroxy groups form dimers in alternating pairs aligned diagonally along the *b* axis and conribute to packing stability (Fig. 2). A view along the *c* axis reveals a hexameric aggregate mediated by a ring of six O—H···O hydrogen bonds generating an R^6^_6_(12) motif loop (Fig. 3).

### Experimental

The title compound was obtained as a gift sample from R. L. Fine Chem, Bengaluru, India. X-ray quality crystals were obtained from benzene solution by slow evaporation. (m.p.: 368–373 K).

### Refinement

H2 was located by a difference map and refined isotropically. All of the remaining H atoms were placed in their calculated positions and then refined using the riding model with Atom—H lengths of 0.95Å or 1.00 (CH), or 0.84° (OH). Isotropic displacement parameters for these atoms were set to 1.2 (CH) or 1.5 (OH) times *U*_eq_ of the parent atom. Idealised tetrahedral OH refined as rotating group: O1(H1).

### Crystal data

**Table:**

C_13_H_11_ClO_2_	*D*_x_ = 1.294 Mg m^−^^3^
*M**_r_* = 234.67	Cu *K*α radiation, λ = 1.5418 Å
Trigonal, *R*3	Cell parameters from 3836 reflections
*a* = 23.4627 (8) Å	θ = 3.8–72.3°
*c* = 11.3722 (4) Å	µ = 2.66 mm^−^^1^
*V* = 5421.6 (4) Å^3^	*T* = 173 K
*Z* = 18	Irregular, colourless
*F*(000) = 2196	0.46 × 0.38 × 0.24 mm

### Data collection

**Table:**

Agilent Xcalibur (Eos, Gemini) diffractometer	2364 independent reflections
Radiation source: Enhance (Cu) X-ray Source	2055 reflections with *I* > 2σ(*I*)
Graphite monochromator	*R*_int_ = 0.047
Detector resolution: 16.0416 pixels mm^-1^	θ_max_ = 72.5°, θ_min_ = 3.8°
ω scans	*h* = −28→25
Absorption correction: multi-scan (*CrysAlis PRO* and *CrysAlis RED*; Agilent, 2012)	*k* = −22→28
*T*_min_ = 0.517, *T*_max_ = 1.000	*l* = −12→13
11532 measured reflections	

### Refinement

**Table:**

Refinement on *F*^2^	Hydrogen site location: inferred from neighbouring sites
Least-squares matrix: full	H atoms treated by a mixture of independent and constrained refinement
*R*[*F*^2^ > 2σ(*F*^2^)] = 0.065	*w* = 1/[σ^2^(*F*_o_^2^) + (0.1357*P*)^2^ + 8.799*P*] where *P* = (*F*_o_^2^ + 2*F*_c_^2^)/3
*wR*(*F*^2^) = 0.213	(Δ/σ)_max_ = 0.001
*S* = 1.07	Δρ_max_ = 0.40 e Å^−^^3^
2364 reflections	Δρ_min_ = −0.62 e Å^−^^3^
151 parameters	Extinction correction: *SHELXL2012* (Sheldrick, 2008), Fc^*^=kFc[1+0.001xFc^2^λ^3^/sin(2θ)]^-1/4^
0 restraints	Extinction coefficient: 0.00038 (12)
Primary atom site location: structure-invariant direct methods	

### Special details

**Table:**

Geometry. All esds (except the esd in the dihedral angle between two l.s. planes) are estimated using the full covariance matrix. The cell esds are taken into account individually in the estimation of esds in distances, angles and torsion angles; correlations between esds in cell parameters are only used when they are defined by crystal symmetry. An approximate (isotropic) treatment of cell esds is used for estimating esds involving l.s. planes.

### Fractional atomic coordinates and isotropic or equivalent isotropic displacement parameters (Å^2^)

**Table:**

	*x*	*y*	*z*	*U*_iso_*/*U*_eq_	
Cl1	0.66585 (5)	0.59567 (4)	0.59620 (10)	0.0780 (4)	
O1	0.64040 (8)	0.41759 (8)	0.77861 (16)	0.0393 (5)	
H1	0.6760	0.4335	0.8159	0.059*	
O2	0.56637 (10)	0.49236 (11)	0.91391 (16)	0.0436 (5)	
H2	0.590 (2)	0.532 (2)	0.888 (4)	0.068 (12)*	
C1	0.63717 (10)	0.46886 (11)	0.71725 (19)	0.0295 (5)	
H1A	0.6573	0.5090	0.7683	0.035*	
C2	0.56447 (11)	0.44658 (11)	0.7019 (2)	0.0310 (5)	
C3	0.53051 (12)	0.45681 (12)	0.7923 (2)	0.0370 (6)	
C4	0.46358 (14)	0.43543 (14)	0.7772 (3)	0.0480 (7)	
H4	0.4400	0.4422	0.8384	0.058*	
C5	0.43171 (13)	0.40498 (16)	0.6757 (3)	0.0541 (8)	
H5	0.3865	0.3918	0.6661	0.065*	
C6	0.46467 (14)	0.39338 (17)	0.5873 (3)	0.0558 (8)	
H6	0.4421	0.3711	0.5177	0.067*	
C7	0.53144 (13)	0.41460 (14)	0.6004 (2)	0.0446 (7)	
H7	0.5544	0.4070	0.5392	0.054*	
C8	0.67533 (10)	0.48591 (12)	0.6030 (2)	0.0329 (5)	
C9	0.69119 (12)	0.54381 (13)	0.5431 (2)	0.0437 (7)	
C10	0.73000 (15)	0.5615 (2)	0.4418 (3)	0.0679 (11)	
H10	0.7409	0.6013	0.4021	0.081*	
C11	0.75243 (16)	0.5216 (3)	0.3997 (3)	0.0783 (14)	
H11	0.7792	0.5339	0.3312	0.094*	
C12	0.73624 (15)	0.4639 (2)	0.4566 (3)	0.0695 (12)	
H12	0.7513	0.4360	0.4264	0.083*	
C13	0.69824 (13)	0.44599 (15)	0.5574 (2)	0.0458 (7)	
H13	0.6876	0.4060	0.5961	0.055*	

### Atomic displacement parameters (Å^2^)

**Table:**

	*U*^11^	*U*^22^	*U*^33^	*U*^12^	*U*^13^	*U*^23^
Cl1	0.0763 (7)	0.0518 (5)	0.1047 (9)	0.0312 (4)	−0.0183 (5)	0.0120 (4)
O1	0.0278 (8)	0.0368 (9)	0.0499 (11)	0.0137 (7)	−0.0014 (7)	0.0132 (7)
O2	0.0534 (11)	0.0509 (12)	0.0340 (9)	0.0318 (10)	0.0080 (8)	−0.0075 (8)
C1	0.0277 (11)	0.0279 (10)	0.0316 (11)	0.0130 (8)	−0.0020 (8)	−0.0008 (8)
C2	0.0282 (11)	0.0317 (11)	0.0341 (11)	0.0158 (9)	0.0014 (8)	0.0026 (9)
C3	0.0403 (13)	0.0361 (12)	0.0416 (13)	0.0243 (10)	0.0078 (10)	0.0066 (10)
C4	0.0406 (14)	0.0513 (16)	0.0607 (17)	0.0294 (12)	0.0158 (12)	0.0136 (13)
C5	0.0283 (12)	0.0592 (18)	0.073 (2)	0.0208 (12)	0.0050 (12)	0.0138 (15)
C6	0.0332 (14)	0.0679 (19)	0.0545 (17)	0.0164 (13)	−0.0109 (12)	−0.0024 (14)
C7	0.0318 (12)	0.0575 (16)	0.0386 (13)	0.0179 (11)	−0.0008 (10)	−0.0053 (11)
C8	0.0229 (10)	0.0350 (12)	0.0339 (12)	0.0093 (9)	−0.0032 (8)	−0.0030 (9)
C9	0.0319 (12)	0.0434 (14)	0.0393 (13)	0.0065 (10)	−0.0076 (10)	0.0084 (10)
C10	0.0386 (15)	0.083 (2)	0.0433 (16)	0.0008 (16)	−0.0057 (13)	0.0225 (16)
C11	0.0346 (15)	0.135 (4)	0.0338 (15)	0.0182 (19)	0.0038 (12)	0.0009 (19)
C12	0.0385 (15)	0.118 (3)	0.0506 (18)	0.0378 (18)	−0.0045 (13)	−0.032 (2)
C13	0.0336 (12)	0.0563 (16)	0.0476 (14)	0.0226 (12)	−0.0031 (10)	−0.0144 (12)

### Geometric parameters (Å, º)

**Table:**

Cl1—C9	1.710 (3)	C5—C6	1.376 (5)
O1—H1	0.8400	C6—H6	0.9500
O1—C1	1.425 (3)	C6—C7	1.394 (4)
O2—H2	0.87 (5)	C7—H7	0.9500
O2—C3	1.617 (3)	C8—C9	1.394 (4)
C1—H1A	1.0000	C8—C13	1.392 (4)
C1—C2	1.524 (3)	C9—C10	1.396 (4)
C1—C8	1.514 (3)	C10—H10	0.9500
C2—C3	1.393 (3)	C10—C11	1.369 (6)
C2—C7	1.383 (4)	C11—H11	0.9500
C3—C4	1.400 (4)	C11—C12	1.372 (7)
C4—H4	0.9500	C12—H12	0.9500
C4—C5	1.367 (5)	C12—C13	1.382 (4)
C5—H5	0.9500	C13—H13	0.9500
			
C1—O1—H1	109.5	C7—C6—H6	120.2
C3—O2—H2	99 (3)	C2—C7—C6	120.5 (3)
O1—C1—H1A	108.1	C2—C7—H7	119.7
O1—C1—C2	106.83 (17)	C6—C7—H7	119.7
O1—C1—C8	111.67 (19)	C9—C8—C1	120.8 (2)
C2—C1—H1A	108.1	C13—C8—C1	121.2 (2)
C8—C1—H1A	108.1	C13—C8—C9	118.0 (2)
C8—C1—C2	113.87 (18)	C8—C9—Cl1	120.2 (2)
C3—C2—C1	119.7 (2)	C8—C9—C10	120.6 (3)
C7—C2—C1	120.6 (2)	C10—C9—Cl1	119.2 (3)
C7—C2—C3	119.7 (2)	C9—C10—H10	119.9
C2—C3—O2	121.7 (2)	C11—C10—C9	120.1 (3)
C2—C3—C4	118.9 (2)	C11—C10—H10	119.9
C4—C3—O2	119.4 (2)	C10—C11—H11	120.1
C3—C4—H4	119.6	C10—C11—C12	119.9 (3)
C5—C4—C3	120.9 (3)	C12—C11—H11	120.1
C5—C4—H4	119.6	C11—C12—H12	119.7
C4—C5—H5	119.8	C11—C12—C13	120.6 (4)
C4—C5—C6	120.4 (2)	C13—C12—H12	119.7
C6—C5—H5	119.8	C8—C13—H13	119.6
C5—C6—H6	120.2	C12—C13—C8	120.7 (3)
C5—C6—C7	119.6 (3)	C12—C13—H13	119.6
			
Cl1—C9—C10—C11	178.1 (2)	C3—C2—C7—C6	0.9 (4)
O1—C1—C2—C3	84.3 (3)	C3—C4—C5—C6	1.6 (5)
O1—C1—C2—C7	−93.8 (3)	C4—C5—C6—C7	−1.8 (5)
O1—C1—C8—C9	−165.2 (2)	C5—C6—C7—C2	0.6 (5)
O1—C1—C8—C13	11.7 (3)	C7—C2—C3—O2	178.5 (2)
O2—C3—C4—C5	−179.8 (2)	C7—C2—C3—C4	−1.2 (4)
C1—C2—C3—O2	0.4 (3)	C8—C1—C2—C3	−151.9 (2)
C1—C2—C3—C4	−179.3 (2)	C8—C1—C2—C7	29.9 (3)
C1—C2—C7—C6	179.0 (3)	C8—C9—C10—C11	0.6 (4)
C1—C8—C9—Cl1	−1.7 (3)	C9—C8—C13—C12	0.8 (4)
C1—C8—C9—C10	175.8 (2)	C9—C10—C11—C12	0.6 (5)
C1—C8—C13—C12	−176.3 (2)	C10—C11—C12—C13	−1.0 (5)
C2—C1—C8—C9	73.7 (3)	C11—C12—C13—C8	0.3 (4)
C2—C1—C8—C13	−109.4 (2)	C13—C8—C9—Cl1	−178.72 (19)
C2—C3—C4—C5	0.0 (4)	C13—C8—C9—C10	−1.2 (4)

### Hydrogen-bond geometry (Å, º)

**Table:**

*D*—H···*A*	*D*—H	H···*A*	*D*···*A*	*D*—H···*A*
O1—H1···O1^i^	0.84	1.84	2.656 (2)	163

Symmetry code: (i) *y*+1/3, −*x*+*y*+2/3, −*z*+5/3.
